# Isolated middle cerebral artery dissection: a systematic review

**DOI:** 10.1186/s12245-014-0044-1

**Published:** 2014-12-17

**Authors:** Ganesh Asaithambi, Pradeepan Saravanapavan, Vaibhav Rastogi, Sheema Khan, Sharatchandra Bidari, Anna Y Khanna, Latha Ganti, Adnan I Qureshi, Vishnumurthy Shushrutha Hedna

**Affiliations:** Department of Neurology, University of Florida College of Medicine, HSC Box 100236, Gainesville, FL 32610 USA; Department of Radiology, University of Florida College of Medicine, Gainesville, FL 32610 USA; North Florida South Georgia Veterans Affairs Medical Center, 1601 Archer Road, Gainesville, FL 32610 USA; Zeenat Qureshi Stroke Institute, 519 2nd St N, St Cloud, MN 56303 USA

**Keywords:** Dissection, Intracranial, Middle cerebral artery, Stroke

## Abstract

Acute stroke can be missed in the emergency department, particularly in younger patients and in those with more vague symptoms such as headache or dizziness. Cervicocephalic dissections are one group of etiologies for acute stroke in the young. While cervicocephalic dissections are not uncommon in clinical practice, isolated middle cerebral artery dissection (MCAD) has been rarely reported as a cause for stroke. We sought to review the clinical implications and pathophysiology of an isolated MCAD. We searched the medical literature for isolated MCAD in clinical stroke patients using MEDLINE, HighWire, and Google Scholar databases from 1966 to 2013 using the keywords ‘middle cerebral artery dissection,’ ‘intracerebral artery dissection,’ and ‘middle cerebral artery dissection stroke.’ We reviewed cases to learn various characteristics of isolated MCAD. A total of 61 cases (62.3% male, mean age 44.16 ± 19.17 years) were reviewed from 54 publications. Most cases were reported from Asian countries (78.7%). Ischemic strokes were more common than hemorrhagic strokes (68.9%). Digital subtraction angiography was the most common imaging modality used to diagnose isolated MCAD (75.4%). Surgery was the preferred form of therapeutic intervention (39.3%). Males (*n* = 27/48, *p* = 0.0008) and those who presented with only ischemic syndromes (*n* = 22/48, *p* = 0.0009) had significantly higher rates of favorable outcome. Isolated MCAD is a rare disease that can contribute to the stroke burden of young patients. Further studies are needed to better characterize optimal treatment strategies and define outcomes for this rare condition.

## Review

### Introduction

Acute neurological deficits are common emergency department presentations with stroke accounting for up to 3.2% of emergency department visits [1]. Isolated middle cerebral artery (MCA) dissection (MCAD) is a rarely reported cause of stroke [2,3]. Although the first case was reported in 1915, isolated MCAD remains poorly understood in comparison to dissections of the intracranial internal carotid artery (ICA) with extension into the MCA. Whether an MCAD occurred with or without ICA dissection, cerebral infarction was the main consequence reported. Given the dynamic process of dissection, it is hypothesized that an initial hemodynamic compromise results from stenosis or occlusion from a lumen wall hematoma with subsequent thrombus formation and distal embolism [4]. A combination of low clinical suspicion and inadequate understanding of the etiology, presentation, imaging findings, and treatment strategies may contribute to the low incidence of isolated MCAD. Therefore, we sought to determine evidence of isolated MCAD through searching the available literature to better understand this unique disease process.

### Methods

A systematic search of MEDLINE, HighWire, and Google Scholar (January 1966 to July 2013) with the keywords ‘middle cerebral artery dissection,’ ‘intracerebral artery dissection,’ and ‘middle cerebral artery dissection stroke’ was conducted in subject headings and keywords. Three reviewers (GA, PS, VSH) independently extracted the data from relevant studies, and discrepancies about inclusion were resolved by VSH. Case reports/series describing characteristics of isolated MCAD were included; cases of MCAD associated with dissections of other vessels were excluded. Articles were assessed for relevancy, and data were compiled for demographics, etiology, symptoms, diagnostic testing, treatment, and outcomes.

Outcomes were dichotomized into favorable and unfavorable, and each case was distributed in either group based on the description provided in the reports. We defined unfavorable outcome as death, modified Rankin Scale (mRS) scores ≥2, or those reported with outcomes as ‘moderate,’ ‘poor,’ or ‘severe morbidity.’ Fisher's exact tests were then used to determine statistical significance of outcomes based on the clinical characteristics collected. Statistical significance was defined as *p* < 0.05. Calculations were performed with SAS software (version 9.4, SAS Institute Inc., Cary, NC, USA).

### Results

Literature review yielded 61 cases (62.3% male, median age 46 [interquartile range 29 to 60] years) from 54 published case reports/series of isolated MCAD (Tables 1 and 2). Cases were described from Asian (78.7%), European (13.1%), and North American (8.2%) countries. No reports from South America, Africa, or Australia fulfilled criteria to be included in our study. Approximately 85.2% of cases were of spontaneous/idiopathic etiology with the remainder occurring from traumatic injury. Common stroke risk factors, including hypertension, diabetes mellitus, dyslipidemia, tobacco abuse, and arrhythmia, were reported in 31.1% of cases. Histories of remote hypertensive intracerebral hemorrhage (ICH) and migraines were also reported.

**Table 1:** **Characteristics of isolated middle cerebral artery dissection by study**

**Case report**	**Age**	**Gender**	**Associated conditions**	**Location**	**Etiology**	**Treatment**
Ischemia						
Piepgras et al. [5]	56	F	-	M1	Idiopathic	S
Sharif et al. [6]	17	M	-	M1	Trauma	C
Adams et al. [7]	12	F	-	-	Trauma	-
Fu et al. [8]	38	M	-	M1	Idiopathic	ET
Gotoh et al. [9]	65	M	-	M2	Idiopathic	-
Fujimura et al. [10]	20	M	Migraine	M1	Idiopathic	S
Kondoh et al. [11]	66	M	Sick sinus syndrome	M1	Idiopathic	AP
Hidaka et al. [12]	31	M	-	M2	Idiopathic	AC
Lin et al. [4]	18	M	-	M1	Trauma	AC
Lee et al. [13]	24	M	-	M1	Idiopathic	AC
	50	F	DM	M1	Idiopathic	AP
Prabhakaran et al. [14]	33	M	-	M1	Trauma	AP + AC
Hsu et al. [15]	15	M	-	M1	Trauma	AP
Kennedy et al. [16]	28	F	-	M1	Idiopathic	AP
Verny et al. [17]	37	F	HTN	M2	Idiopathic	AP
Abe et al. [18]	27	M	Tobacco abuse, asthma	M1	Trauma	S
Han et al. [19]	49	M	-	M1	Trauma	AP
Liu et al. [20]	26	M	-	M1	Idiopathic	AP
Suter et al. [21]	9	F	-	M1	Idiopathic	-
Kato et al. [22]	46	M	Hyperlipidemia	M1	Idiopathic	AC
Naggara et al. [23]	25	M	-	M1	Trauma	-
Aoki et al. [24]	57	M	-	M1	Idiopathic	AP
Watanabe et al. [25]	30	M	-	-	Idiopathic	AP
Lee et al. [26]	56	F	Hyperlipidemia	M1	Trauma	IVT + ET
Torihashi et al. [27]	62	M	-	M1	Idiopathic	S
Yagi et al. [28]	72	F	-	M1	Idiopathic	IVT
Chen et al. [29]	47	M	HTN	M1	Idiopathic	AP
			Tobacco abuse			
Doijiri et al. [30]	45	M	-	M2	Idiopathic	IVT
Kwak et al. [31]	42	M	DM	M1	Idiopathic	AP + AC
	46	F	HTN	M1	Idiopathic	AP + AC
	60	M	HTN	M1	Idiopathic	C
Jung et al. [32]	29	M	-	M1	Idiopathic	-
Iida et al. [33]	53	M	-	M1	Idiopathic	AC
Ischemia + hemorrhage						
Kawaguchi et al. [34]	48	M	-	M2	Idiopathic	S
Hashimoto et al. [35]	56	F	-	M1	Idiopathic	S
Mizutani et al. [36]	67	F	-	M1	Idiopathic	-
Abiko et al. [37]	33	F	-	M1	Idiopathic	C
Niikawa et al. [38]	46	F	-	M1	Idiopathic	S
Esposito et al. [39]	69	M	HTN	M3	Idiopathic	S
			DM			
Yakushiji et al. [40]	40	M	HTN	M1	Idiopathic	C
Hemorrhage						
Sasaki et al. [41]	41	F	-	M2	Idiopathic	S
Chang et al. [42]	73	M	HTN	M2	Idiopathic	C
Mizutani et al. [36]	41	F	-	M1	Idiopathic	S
Abiko et al. [37]	59	F	-	M1	Idiopathic	S
Bosch et al. [43]	68	M	HTN, previous ICH, gout, tobacco abuse	M1	Idiopathic	-
Nimura et al. [44]	61	F	-	M1	Idiopathic	S
Nakashima et al. [45]	63	F	HTN	M2	Idiopathic	S
Ono et al. [46]	68	F	-	M1	Idiopathic	-
Nakashima et al. [47]	29	M	-	M2	Idiopathic	S
Isono et al. [48]	30	M	-	M1	Idiopathic	S
Sakamoto et al. [49]	65	F	-	M3	Idiopathic	S
Ning et al. [50]	50	M	-	M1	Idiopathic	S
Shioya et al. [51]	64	M	-	M2	Idiopathic	S
Peron et al. [52]	1.66	-	-	M1	Idiopathic	S
	8	M	-	M1	Idiopathic	S
	20	M	-	M1	Idiopathic	S
Saito et al. [53]	59	F	-	M1	Idiopathic	S
Chuang et al. [54]	79	M	HTN	M2	Idiopathic	S
Oyama et al. [55]	70	F	-	M2	Idiopathic	S
Transient ischemic attack						
Iwamuro et al. [56]	57	M	-	M1	Idiopathic	AP
Horie et al. [57]	37	M	-	M2	Idiopathic	-

AC, anticoagulation; AP, antiplatelet; C, conservative (conservative treatment indicates medical treatment but unclear if anticoagulant/antiplatelet therapy was included); DM, diabetes mellitus; ET, endovascular treatment; HTN, hypertension; ICH, intracerebral hemorrhage; IVT, intravenous thrombolysis; S, surgery.

**Table 2:** **Demographic and clinical characteristics of isolated middle cerebral artery dissection**

	**Value**
Age (years)	
Median [IQR]	46 [29-60]
<45	28 (45.9%)
45 to 59	17 (27.9%)
>60	16 (26.2%)
Gender (*n* = 60)	
38 (62.3%) male	
Location (*n* = 60)	
M1	45 (75%)
M2	13 (21.7%)
M3	2 (3.33%)
Syndrome (*n* = 61)	
Ischemia	33 (54.1%)
Hemorrhage	19 (31.1%)
Ischemia + hemorrhage	7 (11.5%)
Transient ischemic attack	2 (3.3%)
Treatment (*n* = 56)	
Surgery	24 (42.9%)
AP	11 (19.6%)
AC	5 (8.9%)
AP + AC	3 (5.4%)
IVT	3 (5.4%)
ET	1 (1.7%)
Conservative	9 (16.1%)

Reported based on available information from publications reviewed. AC, anticoagulation; AP, antiplatelet; ET, endovascular treatment; IVT, intravenous thrombolysis.

#### Presenting symptoms and signs

Presenting symptoms included headache (44.3%), seizure (6.6%), nausea/vomiting (4.9%), and tinnitus (1.6%). Depending on laterality, neurologic deficits upon presentation were associated with those commonly found in MCA lesions: weakness (52.5%), changes in speech (34.3%), alterations in level of consciousness (27.9%), numbness (6.6%), or visual field deficits (1.6%). Patients presenting without any observed neurological deficits were also common (19.7%).

#### Types of strokes

A majority of the cohort studied presented with ischemic stroke syndromes (68.9%). Approximately 54.1% of patients developed ischemic strokes only, 11.5% developed concurrent ischemic and hemorrhagic strokes, and 3.3% developed transient ischemic symptoms. The remaining 31.1% of reported patients developed either ICH or subarachnoid hemorrhage (SAH). All traumatic MCADs resulted in ischemic syndromes only.

Approximately 37.7% of patients had aneurysms associated with MCADs. More than half of these patients developed hemorrhagic strokes (13 hemorrhage only, 4 hemorrhage and ischemia). Five patients were found to have ischemic lesions, and one patient developed transient ischemic symptoms.

#### Diagnostic modalities

The most commonly used imaging modality for diagnosis was digital subtraction angiography (DSA, 75.4%) followed by computed tomography/angiography (CT/CTA, 72.1%), magnetic resonance imaging/angiography (MRI/MRA, 62.3%), pathologic evaluation/autopsy (9.8%), and transcranial Doppler (TCD, 1.6%). M1 dissections were reported in 75% of cases, and even more distal dissections (M2 or M3 segments) had higher rates of concurrent aneurysms (10/15 cases).

#### Treatment approaches

Surgery (craniotomy with trapping, wrapping, clipping, extracranial-intracranial bypass, or resection) was the preferred form of therapeutic intervention (39.3%). Intravenous (IV) thrombolytic and endovascular therapies were utilized in only a small minority of cases among those with ischemic syndromes only. Among ischemic strokes only (*n* = 33), long-term antiplatelet therapy (30.3%) was preferred over anticoagulation (15.2%); however, in 9.1% of cases, antiplatelet therapy and anticoagulation therapy were combined. No preventative strategy was described in 8.2% of cases, and in another 14.8% of cases, ‘conservative strategies’ were described but did not distinguish between antithrombotic use or not. Among patients with SAH/ICH, six patients did not undergo surgical treatment (three due to death, and three were conservatively managed). Only four patients who presented with isolated ischemic strokes underwent surgery.

#### Outcomes

Many cases reviewed for our study used subjective terms to describe patients' outcomes. These terms included ‘good,’ ‘stable,’ ‘mild,’ ‘able to ambulate,’ ‘improved,’ ‘independent,’ ‘stable,’ ‘no change,’ ‘moderate,’ ‘poor,’ and ‘severe morbidity.’ Very few cases (8.2%) utilized objective measures including the National Institutes of Health Stroke Scale (NIHSS) or mRS to define outcomes. Approximately 18% of cases did not report outcomes. Of the 48 cases reporting outcomes, 15 patients (mean age 44.64 ± 22.43 years) had unfavorable outcomes (Table 3). Mortality occurred in 6 of the 15 patients with unfavorable outcomes (mean age 48.5 ± 26 years). Males (*n* = 27/48, *p* = 0.0008) and those who presented with only ischemic syndromes (*n* = 22/48, *p* = 0.0009) had significantly higher rates of favorable outcome. The location of the dissection did not appear to affect the outcome (*p* = 0.7898). Patients who were treated with antiplatelet or anticoagulant therapy had significantly higher rates of favorable outcome (*p* = 0.0017). By grouping age of patients by tertiles (<45 years, 45 to 59 years, >60 years), no significant differences in outcomes based on age were found (0.3894).

**Table 3:** **Outcomes based on clinical characteristics reported**

**Clinical characteristic**		**Favorable outcome**	**Unfavorable outcome**
Age (years, *n* = 48)	<45	14 (29.2%)	6 (12.5%)
	45 to 59	12 (25%)	3 (6.3%)
	>60	7 (14.5%)	6 (12.5%)
Gender (*n* = 47)	Female	6 (12.8%)	10 (21.3%)
	Male	27 (57.4%)	4 (8.5%)
Location (*n* = 47)	M1	25 (53.2%)	12 (25.5%)
	M2	7 (14.9%)	2 (4.3%)
	M3	1 (2.1%)	0
Condition (*n* = 48)	None	1 (2.1%)	0
	Ischemic	22 (45.8%)	2 (4.2%)
	Hemorrhage	8 (16.7%)	8 (16.7%)
	Ischemia + hemorrhage	2 (4.1%)	5 (10.4%)
Treatment (*n* = 48)	Antiplatelet	10 (20.8%)	0
	Anticoagulation	3 (6.3%)	0
	Antiplatelet + anticoagulation	3 (6.3%)	0
	Surgery	13 (27.1%)	8 (16.7%)
	Intravenous thrombolysis	2 (4.1%)	0
	Conservative	2 (4.1%)	7 (14.6%)

### Discussion

Based on our review, isolated MCAD affects a younger cohort of patients as compared to those who suffer from strokes due to traditional etiologies. Headache appears to be the commonest complaint of the predominantly spontaneous condition. While most patients do not present with focal neurologic abnormalities, those who do present with such have common deficits based on location of the MCA territory involved.

Cervicocephalic dissections have been ascribed as a complication of blunt or penetrating trauma; however, spontaneous dissections may be as, or even more, common [3]. The M1 segment of the MCA appears to be most commonly involved in MCAD due to its proximity to the posterior margin of the sphenoid wing; with friction between the M1 segment and this bony anatomic landmark, injury may occur [4,12,13,15,19,27].

There are many associated conditions that may predispose MCAD (or dissections of other cerebral arteries). These include migraine, fibromuscular dysplasia, cystic medial necrosis, intimal fibroelastic irregularities, homocystinuria, periarteritis nodosa, syphilitic arteriopathy, moyamoya disease, atherosclerosis, Guillain-Barre syndrome, Marfan's syndrome, and Ehlers-Danlos syndrome [2-6,13,18,35,58,59]. Further, protein expression and activation have been shown to cause occlusion, arterial wall fragility, and rupture of the aneurysm [53].

Dissections, particularly when associated with aneurysms, of the anterior circulation typically manifest as ischemic syndromes, but similar dissections of the posterior circulation usually manifest as SAH [31,36,41]. This postulation was based on previously described classifications of intracranial dissecting aneurysms. A type 1 dissecting aneurysm occurs between the internal elastic lamina and the media, causing ischemia from occlusion or stenosis. On the other hand, a type 2 dissecting aneurysm results in SAH due to dissection between the media and the adventitia. Type 2 dissecting aneurysms are commonly found in the posterior circulation, which is why this system has a propensity for SAH [38,41,49]. Our review suggested otherwise in that more than half of the patients with aneurysms associated with MCAD developed ICH and/or SAH. When not associated with an aneurysm, however, the rate of ischemic syndromes associated with MCAD was higher.

Both invasive and noninvasive methods have been utilized to detect MCAD. These modalities include CT/CTA, MRI/MRA, DSA, TCD, and pathologic evaluation. While it was previously reported that intracranial dissections are more commonly diagnosed post-mortem, our review of isolated MCAD cases revealed only one report confirming MCAD at the time of autopsy only [2,7,15]. DSA is the gold standard for identifying cervicocephalic arterial dissections [13,20,29]. However, the use of DSA may not always demonstrate an intimal flap, false lumen, or pseudoaneurysms, which are common entities within a dissection; instead, it commonly can show segmental stenosis of the involved vessel segment [4,19,20]. In the setting of a rare condition, intracranial segmental stenoses could lead to an incorrect diagnosis.

**Figure 1:**
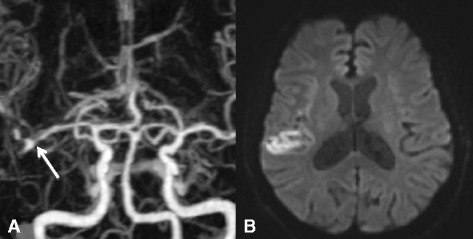
**Noninvasive imaging. (A)** Three-dimensional 360-slice CTA of the head of a 63-year-old woman presenting with headaches after a motor vehicle collision demonstrating a right M1 dissection with pseudoaneurysm immediately before the bifurcation (arrow); there is normal caliber of the contralateral MCA. **(B)** MRI brain shows diffusion restriction within the right perisylvian region consistent with acute ischemia. She was discharged on antiplatelet therapy with a modified Rankin Scale score of 0.

**Figure 2:**
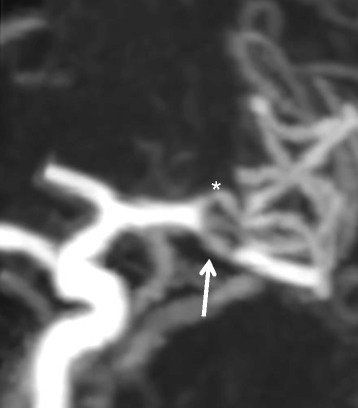
**Noninvasive imaging.** Three-dimensional 360-slice CTA of the head of a 50-year-old man presenting with abrupt-onset 2-day history of headache and right hemiparesis demonstrating left M1 dissection (arrow) distal to the anterior temporal branch (asterisk). He was discharged on antiplatelet therapy with a modified Rankin Scale score of 0.

Noninvasive imaging may be useful, if not superior based on the modality used, as adjunctive diagnostic testing (Figures 1 and 2). High-resolution MRI (HRMRI) was described as an effective noninvasive means to diagnose isolated MCAD based on its ability to pay close attention to the structural characteristics of the vessel wall and lumen [23,26]. Kwak et al. later confirmed the utility of HRMRI by successfully using three-dimensional magnetization-prepared rapid acquisition gradient-echo sequences to detect intimal flaps of dissections and concurrent intramural hemorrhages in their case series [31]. Kato et al. described a method of using standard T2-weighted MRI sequencing in the coronal plane parallel to the sylvian vallecula with gray-scale reversal during post-processing that is superior to MRA in detecting MCAD. Yet they note that this anatomically based imaging method is limited to the horizontal segment of the MCA [22]. By an even more crude method, Jung et al. noted a ‘shadow sign’ on axial T2-weighted MRI sequences, which can suggest intramural hematoma and clinch the diagnosis of dissection; Chen et al. described a similar method as well [29,32]. However, invasive and noninvasive neuroradiologic studies can be limiting and may not be sufficient in detecting MCAD. Some reports have described detection of MCAD with associated aneurysmal rupture after patients underwent craniotomy for hematoma evacuation. Once discovered, these lesions were surgically repaired [45,47,51].

The best method of therapeutic intervention for isolated MCAD remains unclear. The treatment strategies included use of antiplatelet agents, anticoagulation, thrombolysis, surgery, endovascular therapy, and no treatment/conservative therapy. While the safety of IV thrombolysis for acute ischemic stroke secondary to extracranial carotid artery dissection has been studied, the safety of thrombolytic use for intracranial dissections remains unknown [60,61]. Doijiri et al. reported successful use of IV recombinant tissue plasminogen activator, albeit at a lower than recommended dose of 0.6 mg/kg, without any clear complications [30]. Further studies on the safety and outcomes with the use of IV thrombolysis in acute ischemic stroke from intracranial dissection are needed. There is trepidation with the use of anticoagulation for isolated MCAD-related ischemic syndromes as it may promote progression of intramural hematoma within the dissection [5], whereas others hypothesize that anticoagulation may slow the progression of thrombosis that could otherwise lead to a fusiform aneurysm [17]. Endovascular therapies were not abundant in our review, and clinicians may be reluctant to use it beyond the acute setting. While Lee et al. described a successful case of MCAD stenting, stenting may pose a risk to occluding MCA perforators [26,27].

The outcome of strokes secondary to isolated MCAD remains to be established with more objective data. Men and those who have ischemic syndromes appear to have higher rates of favorable outcome based on subjective descriptions. Age did not appear to have a significant effect on outcomes. The benefit of surgery remains unclear, as those who were already at risk for a worse outcome due to hemorrhage were the ones who more commonly underwent surgery. The utility of IV thrombolysis or endovascular therapy requires further evaluation.

While this study is likely the most comprehensive review of isolated MCAD and its clinical implications, it has important limitations. Approximately 23% of cases reported were written in alternate languages with only abstracts available in English. With review of abstracts only, key characteristics of reported patients are missed. While most reported cases were from Asian countries, to suggest that Asians are more susceptible to isolated MCAD would introduce a selection bias. Formal outcome scales were rarely used, including the NIHSS or mRS scores. Therefore, our definition of unfavorable outcomes was based on subjective descriptions. Without standardized outcome definitions, the best therapeutic interventions remain unknown, as it was based on expert opinion case-by-case. Prospective studies are needed to define outcomes based on therapeutic interventions.

## Conclusions

Isolated MCAD remains an underreported cause of stroke that affects a younger population than commonly seen by traditional etiologies of stroke. While more likely to result in ischemic strokes, hemorrhagic strokes can occur especially when associated with aneurysms. Isolated MCAD is most likely to be idiopathic/spontaneous in etiology and affects a younger population. Noninvasive diagnostic studies are capable of detecting this condition when there is a high index of clinical suspicion. Optimal therapeutic interventions remain unclear, and unfavorable outcomes are higher among those who have hemorrhagic strokes. Further studies are needed to better characterize optimal treatment strategies that give patients the highest chance of favorable outcomes.
